# Ionothermal Synthesis of Sulfidobismuth *spiro*‐Dicubane Cations

**DOI:** 10.1002/open.202100145

**Published:** 2021-08-13

**Authors:** Maximilian Knies, Michael Ruck

**Affiliations:** ^1^ Faculty of Chemistry and Food Chemistry Technische Universität Dresden 01069 Dresden Germany; ^2^ Max Planck Institute for Chemical Physics of Solids Nöthnitzer Str. 40 01187 Dresden Germany

**Keywords:** bismuth, heterocubanes, ionic liquids, ionothermal synthesis, spirocubanes

## Abstract

Bi_2_S_3_ was dissolved in the presence of NaCl in the ionic liquid [BMIm]Cl ⋅ 4AlCl_3_ (BMIm=1‐*n*‐butyl‐3‐methylimidazolium) through annealing the mixture at 180 °C. Upon cooling to room temperature, orange, air‐sensitive crystals of Na(Bi_7_S_8_)[S(AlCl_3_)_3_]_2_[AlCl_4_]_2_ (**1**) precipitated. X‐ray diffraction on single‐crystals of **1** revealed a triclinic crystal structure that contains (Bi_7_S_8_)^5+^ *spiro*‐dicubanes, [S(AlCl_3_)_3_]^2−^ tetrahedra triples, isolated [AlCl_4_]^−^ tetrahedra, and sodium cations.

## Graphical abstract

**In an ionothermal synthesis** bismuth sulfide reacted to *spiro*‐dicubane cations (Bi_7_S_8_)^5+^, which crystallized in a complex structured salt. Diverse alkali metal cations were incorporated alongside these polycations, hinting at the existence of a structure family, despite stacking faults preventing a full structure elucidation of all compounds.

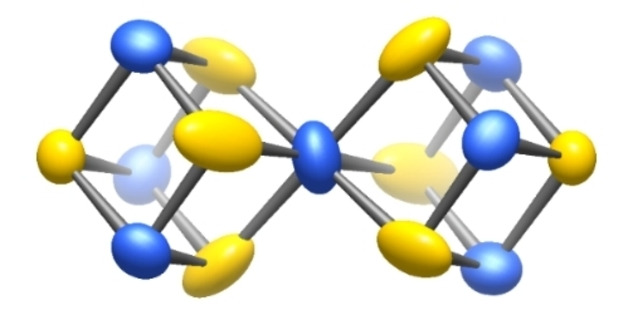

## 1 Introduction

The use of ionic liquids (ILs)^[1]^ as solvents in inorganic synthesis is becoming increasingly popular as they make materials accessible at low temperatures and in high purity or open paths to new compounds that cannot be synthesized by conventional methods.[^2^, ^3^, ^4^, ^5^, ^6^, ^7^, ^8^, ^9^, ^10^, ^11^, ^12^, ^13^] Some ILs are able to dissolve inorganic compounds that are conventionally hardly soluble without harsh conditions.[^14^, ^15^] One example is bismuth(III) sulfide, Bi_2_S_3_, also known as the mineral bismuthinite, which usually requires conditions such as hot nitric acid to be dissolved but readily does so in ILs.[^16^, ^17^, ^18^] The formation of complex ionic species seems to be facilitated in ILs through incorporation of and stabilization through weakly coordinating anions, originating from the solvent itself.[^6^, ^10^, ^13^] Thus, contrary to most other solvents, ILs cannot always be regarded as inert reaction media.[^19^, ^20^, ^21^]

Salts containing cations composed of pnicogen and chalcogen atoms are comparatively rare. In 1975, Gillespie et al. reported the first cationic sulfidonitrogen rings.[^22^, ^23^, ^24^] The up to now solitary binary cation containing phosphorous, the polyhedral (P_3_Se_4_)^+^, was discovered only recently.^[25]^ For the semi‐metallic elements of group 15, the isostructural cages (As_3_ *Ch*_4_)^+^ (*Ch*=S, Se) are known.[^26^, ^27^] The sulfur‐containing cation also occurs with a capping atom as (As_3_S_5_)^+^.^[27]^ Reactions with tellurium led to the trigonal prism (As_2_Te_4_)^2+^.^[28]^ Antimony‐based pnicogen‐chalcogen polycations exist in great variety of shapes and sizes, e. g. the (Sb_2_Te_4_)^2+^ prism,^[28]^ the capped trigonal prism (Sb_3_Te_4_)^3+^,^[28]^ the cubes (Sb_4_ *Ch*_4_)^4+[28]^ or the *spiro*‐dicubanes (Sb_7_ *Ch*_8_)^5+^ with *Ch*=S, Se, Te. The latter crystallized without[^29^, ^30^] or together with other metal cations,[^30^, ^31^] or as (Sb_7_ *Ch*_8_ *X*_2_)^3+^ with terminal halogen atoms (*X*=Cl, Br).[^30^, ^32^, ^33^] Furthermore, *spiro*‐tetracubanes [Sb_13_Se_16_Br_2_]^5+^,^[32]^ linked realgar cages [Sb_10_Se_10_]^2+^,^[34]^ and periodic *catena*‐polycations[^35^, ^36^] were isolated.

Cuboid shapes are also dominating among the bismuth‐chalcogen cations. *Beck* et al. crystallized (Bi_4_S_4_)^4+^, the first binary sulfidobismuth(III) polycation, from a NaAlCl_4_ flux.^[37]^ Heterocubanes were also observed for the heavier chalcogen homologues in (Bi_4_ *Ch*_4_)[*Tr*Cl_4_]_4_ (*Ch*=Se, Te, *Tr*=Al, Ga).[^28^, ^37^, ^38^] Substitution of one bismuth vertex with an Al−Cl^[17]^ or a Ga−S^[39]^ dumbbell as well as extension to a *spiro*‐dicubane^[18]^ were observed so far. We recently discovered the double salt [BMIm](Bi_4_S_4_)[AlCl_4_]_5_ (BMIm=1‐*n*‐butyl‐3‐methylimidazolium), which incorporates the reaction medium [BMIm][AlCl_4_] and precipitates only in the presence of metal cations like gold(I) or platinum(II).^[18]^

The incorporation of metal cations alongside *spiro*‐dicubane polycations has particularly been observed for the isostructural telluridoantimony(III) compounds *M*(Sb_7_Te_8_)[*Tr*Cl_4_]_6_ (*M*=Ag, Cu, Na; *Tr*=Al, Ga).[^29^, ^30^, ^31^] The sodium and silver compounds undergo phase transitions between 177 K and 93 K, which leads to splitting of the monovalent metal cation positions accompanied by a doubling of the lattice parameter *c*.[^29^, ^30^] Besides, only the recently discovered Ag(Bi_7_S_8_)[S(AlCl_3_)_3_]_2_[AlCl_4_]_2_ contains binary pnicogen‐chalcogen cations alongside metal ions.^[18]^ Its triclinic crystal structure is a stack of layer packages with trigonal layer symmetry. The pseudosymmetry is induced by the molecular symmetry of the *spiro*‐dicubane and the [S(AlCl_3_)_3_]^2−^ anion. It provides numerous positions of similar energy for the silver cations, which results in extended disorder that persists even upon slow cooling of the crystals.

Herein, we report on the IL‐based synthesis and crystal structure of a new sulfidobismuth(III) compound, incorporating alkali metal ions, Na(Bi_7_S_8_)[S(AlCl_3_)_3_]_2_[AlCl_4_]_2_ (**1**) as well as experimental indications of similar compounds containing potassium, rubidium, or cesium.

## 2 Results and Discussion

### 2.1 Synthesis Results and Substitution Attempts

A mixture of NaCl and Bi_2_S_3_ in the molar ratio of 2 : 1 was dissolved in the ionic liquid [BMIm]Cl ⋅ 4AlCl_3_ at 180 °C. Upon cooling to room temperature, deep red, shiny crystals of Na(Bi_7_S_8_)[S(AlCl_3_)_3_]_2_[AlCl_4_]_2_ (**1**) crystallized alongside minor amounts of [BMIm](Bi_4_S_4_)[AlCl_4_]_5_ ^[18]^ and solidified AlCl_3_ (Figure S1, Supporting Information). Energy dispersive X‐ray spectroscopy (EDX) confirmed the composition, particularly the presence of sodium in **1**.

Analogous syntheses using the chlorides of other alkali metals (*M*=K, Rb, Cs) yielded crystals of similar color, shape and composition, according to EDX measurements. X‐ray diffraction experiments on these crystals showed a high degree of diffuse scattering on the reflection rows parallel the b* axis, as well as low Bragg intensities. We suppose stacking faults to cause these effects, which also affect the crystal structure of **1** but to a lesser extent. Although a full crystal structure analysis was impossible, at least approximate lattice parameters for the compounds with *M*=K, Cs could be determined (Table 1). These unit cells resemble the one of Ag(Bi_7_S_8_)[S(AlCl_3_)_3_]_2_[AlCl_4_]_2_,^[18]^ strongly suggesting compounds *M*(Bi_7_S_8_)[S(AlCl_3_)_3_]_2_[AlCl_4_]_2_ with analogous layered structures.

**Table 1:** Lattice parameters of compounds *M*(Bi_7_S_8_)[S(AlCl_3_)_3_]_2_[AlCl_4_]_2_ with *M*=Ag, K, Cs at room temperature.

*M*	Ag^[16]^	K	Cs
a/pm	1064.5(1)	1052(18)	1068(2)
b/pm	1067.0(1)	1061(20)	1068(2)
c/pm	1513.3(1)	1560(20)	1608(3)
α/°	77.17(1)	77.8(4)	70.7(1)
β/°	75.59(1)	70.8(4)	74.4(1)
γ/°	60.42(1)	61.2(4)	60.1(1)
V/(10^6^ pm^3^)	1437.7(1)	1438(8)	1488(1)

### 2.2 Crystal Structure of Na(Bi_7_S_8_)[S(AlCl_3_)_3_]_2_[AlCl_4_]_2_

**Figure 1:**
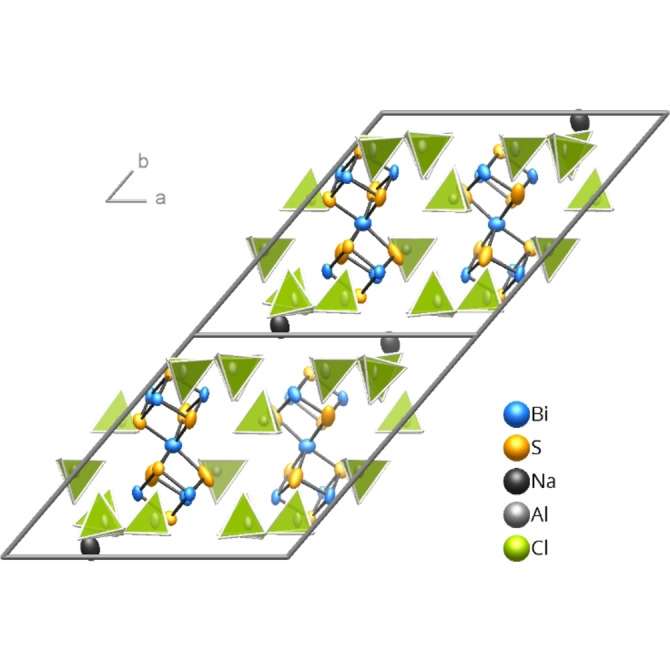
Crystal structure of **1** at 100(2) K. [S(AlCl_3_)_3_]^2−^ and [AlCl_4_]^−^ anions are depicted as Al‐centered polyhedra. The ellipsoids comprise 99 % of the probability density of the atoms.

**Figure 2:**
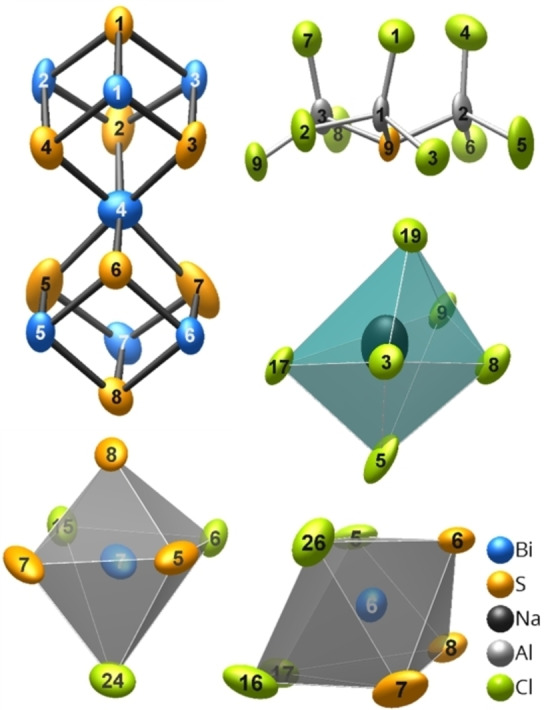
(Bi_7_S_8_)^5+^ *spiro*‐dicubane cation, [S(AlCl_3_)_3_]^2−^ anion, and coordination polyhedra of the sodium(I) cation and bismuth(III) atoms in **1**. The ellipsoids comprise 99 % of probability density of atoms at 100(2) K.

Sodium(I)‐*spiro*[8,8]sulfidobismuth(III)cubane‐bis{tris[trichloridoaluminate(III)]sulfide}‐bis[tetrachloridoaluminate(III)], **1**, crystallizes in the triclinic space group *P* $\overset{-}{1}$ (no. 2) with two formula unit per unit cell and the lattice parameters *a*=1846.4(2) pm, *b*=1905.1(2) pm, *c*=1065.5(1) pm, *α*=84.07(1)°, *β*=89.55(1)°, *γ*=49.68(1)° and *V*=2833.9(4) 10^6^ pm^3^ at 100(2) K. Atomic parameters and interatomic distances are listed in Tables S1 and S2 of the Supporting Information. The crystal structure (Figure 1) features three different complex ions, (Bi_7_S_8_)^5+^ *spiro*‐dicubane cations, [S(AlCl_3_)_3_]^2−^ anions and [AlCl_4_]^−^ tetrahedra, as well as sodium(I) cations (Figure 2).

The structure of **1** is composed of layer packages (*d*≈1160 pm), which resemble those in Ag(Bi_7_S_8_)[S(AlCl_3_)_3_]_2_[AlCl_4_]_2_ (**2**),^[18]^ and Na^+^ cations in the inter‐layer space (*d*≈260 pm). While the silver compound **2** has a high layer symmetry that was reduced to *P* $\overset{-}{1}$ through stacking, in the sodium compound **1**, the molecular units themselves are distorted and the deviations from the layer group *p* $\bar{3}$ *m*1 (no. 72)^[40]^ are larger than in **2**. This goes hand in hand with an ordered distribution of the monovalent cations in **1** and seems to be associated with the size and the polarizability of the *M* ^*+*^ cation. Among the discussed monovalent cations, Na^+^ is the smallest and hardest (Shannon radii for sixfold coordination in pm: Na 102, Ag 115, K 138, Rb 152, Cs 167).^[41]^

The unit cell of the sodium compound **1** is twice as large as that of the silver compound **2**. Conventions for the setting of unit cells make *b* the stacking vector for **1**, while it is *c* for **2**. The thickness of the layer packages in both compounds differ only by few percent. The bases of the unit cells that are parallel to the layers are also related: While in **2**, the *a* and *b* vectors define a rhombic grid, following the trigonal layer symmetry, the vectors *a* and *c* of **1** describe a rectangle, which corresponds to an orthohexagonal setting (*a* – *c* $\sqrt{3}$ =0.9 pm), yet without centering. The reflections *h*+*l*=2*n*+1, which violate the reflection conditions for the *B* centering and define the larger unit cell for **1**, are much weaker than those with even index sums. In the crystal structure of **1** (Figure 1), the different *y* coordinates of the Na^+^ cations in the same layer interspace are the most obvious deviations from the *B* centering. If the disordered Ag^+^ distribution in **2** is neglected, the structure of **1** can be seen as crystallizing in a *klassengleiche* sub‐group of index 2 of the space group of compound **2**.^[42]^ A representation of the structure of **2** in the “orthohexagonal” setting can be found in the Supporting Information (Figure S2). The positions of the *spiro*‐atoms in **1** and **2** (Table S3) deviate only by Δ*x*=0.014, Δ*y*=0.002, and Δ*z*=−0.020, supporting the close relation between the two structures.

The hetero *spiro*‐dicubane (Bi_7_S_8_)^5+^ (Figure 2) is isomorphous to the cations (Sb_7_Se_8_)^5+^ and (Sb_7_Te_8_)^5+^.[^29^, ^30^, ^31^] It is composed of two (Bi_4_S_4_)^4+^ cubes that share a common bismuth atom vertex. An idealized (Bi_7_S_8_)^5+^ molecule would have the point group symmetry $\overset{-}{3}$ *m* (*D*_3d_). However, the observed crystallographic symmetry is only 1 (*C*_1_), even lower than in Ag(Bi_7_S_8_)[S(AlCl_3_)_3_]_2_[AlCl_4_]_2_, where the central bismuth atom occupies the 1*a* Wyckoff position, representing the molecular symmetry $\overset{-}{1}$ (*C*_i_).^[18]^ While in **2**, the *spiro*‐atom is in an almost regular octahedral environment [280.9(4)–281.4(3) pm],^[18]^ its counterpart in **1** (Bi4) has a [3+3] coordination with three shorter [271.5(4)–272.4(4) pm] and three longer [288.2(4)–292.6(4) pm] Bi–S bonds. For the threefold coordinated bismuth atoms the range of Bi–S distances is also broader in **1** [252.5(4)–266.7(3) pm] than in **2** [259.1(3)–267.2(2) pm].^[18]^ These bismuth atoms are additionally coordinated by chloride ions of the [AlCl_4_]^−^ and [S(AlCl_3_)_3_]^2−^ groups, with Bi⋅⋅⋅Cl distances ranging from 304.4(3) to 352.3(3) pm. Bi7 has a distorted octahedral [3S+3Cl] coordination, while the other bismuth atoms reside in distorted, capped trigonal prisms formed by [3S+4Cl] (Figure 2). The differences that the interatomic distances of the (Bi_7_S_8_)^5+^ cation show in two comparatively similar compounds demonstrate its sensitivity to the chemical environment. Thus, a detailed comparison to the *spiro‐*dicubanes in *M*(Sb_7_Te_8_)[*Tr*Cl_4_]_6_ (*M*=Ag, Cu, Na; *Tr*=Al, Ga)[^29^, ^30^, ^31^] appears to be not very instructive.

The Na^+^ cation is octahedrally coordinated by six chloride ions that belong to four [S(AlCl_3_)_3_]^2−^ anions and one [AlCl_4_]^−^ tetrahedron. The Na⋅⋅⋅Cl distances [280.6(7)–306.0(7) pm] match those observed in Na[AlCl_4_],^[43]^ but are longer than in NaCl (281 pm). The displacement ellipsoid of the Na^+^ cation is remarkably large, which might be associated with stacking faults (see below).

The two isolated [AlCl_4_]^−^ tetrahedra show significant differences. The tetrahedron around Al7 is almost regular with bond lengths ranging from 213.0(5) pm to 213.9(5) pm, while the Al8 polyhedron shows four slightly deviating distances between 212.0(5) pm and 215.6(5) pm. All Al−Cl distances are in accordance with those observed in Na[AlCl_4_]^[43]^ and their variation can be correlated with secondary Cl⋅⋅⋅Bi or Cl⋅⋅⋅Na bonds.

The anionic group [S(AlCl_3_)_3_]^2−^ consists of three [AlSCl_3_]^2−^ tetrahedra that share their sulfur vertex (Figure 2). All polyhedra point in the same direction. The pseudo‐symmetry is 3 *m* (*C*_3v_), while the crystallographic symmetry is only 1 (*C*_1_). Despite the tilting of the tetrahedra, the S−Al bond lengths are rather uniform [226.5(5)–228.2(5) pm]. This anion was first observed in (Bi_3_S_4_AlCl)[S(AlCl_3_)_3_][AlCl_4_].^[17]^ Most likely a gallium analogue exists in (Bi_3_GaS_5_)[Ga_3_Cl_10_]_2_[GaCl_4_]_2_⋅S_8_,^[39]^ assuming some erroneous assignment of S and Cl, which have very similar scattering factors. Moreover, the selenium compound (Bi_4_Se_4_)[Se(GaCl_3_)_3_][GaCl_4_] exists, which corroborates the atom assignment.^[28]^

All investigated crystals of **1** suffered from stacking disorder that caused streaks of diffuse scattering on the reflection rows parallel to b*. The stacking disorder, or twinning in the case of large domains, is a consequence of the trigonal pseudosymmetry of the layer packages, which permits a rotation of the stacking vector by ±120°. Because of the translational pseudosymmetry, represented by the above‐mentioned group‐subgroup relationship, antiphase boundaries can be expected in addition. Although the selected crystal appeared to be relatively unaffected by stacking faults, its real structure manifested itself as unusually high residual electron densities, especially in the immediate vicinity of the bismuth atoms.

## 3 Conclusions

Lewis‐acidic ionic liquids that contain an excess of AlCl_3_ proved their ability to activate crystalline Bi_2_S_3_ at moderate temperatures.

Although the IL is a non‐oxidizing solvent, it can replace hot nitric acid, which is commonly used for this purpose. Together with NaCl and the anionic part of the IL, the dissolved Bi_2_S_3_ forms the complex structured salt Na(Bi_7_S_8_)[S(AlCl_3_)_3_]_2_[AlCl_4_]_4_. Its layered structure includes cationic *spiro*‐dicubanes (Bi_7_S_8_)^5+^ and anionic tetrahedra triples [S(AlCl_3_)_3_]^2−^. Analogous syntheses using the heavier alkali metal ions *M* ^+^ (*M*=K, Rb, Cs) yielded compounds of the same kind, whose crystal structures, however, could not be determined because of extensive stacking disorder. The method is not limited to Bi_2_S_3_ and can also be used to activate other (sulfidic) minerals. Similar ionometallurgical approaches^[44]^ have the potential to substitute conventional processes, such as roasting of sulfidic ores, which are associated with high temperatures and the formation of gases with high environmental impact.

## Experimental Section

**Synthesis**: All compounds were handled in an argon‐filled glove box (MBraun; *p*(O_2_)/*p* ^0^<1 ppm, *p*(H_2_O)/*p* ^0^<1 ppm). The reactions were carried out in silica ampules with a length of 120 mm and a diameter of 14 mm. Na(Bi_7_S_8_)[S(AlCl_3_)_3_]_2_[AlCl_4_]_2_ was synthesized in the ionic liquid [BMIm]Cl ⋅ 4AlCl_3_, which acted as solvent and reactant. The ampule was loaded with 23.4 mg NaCl (0.4 mmol, 99.98 %, Alfa Aesar), 104.0 mg Bi_2_S_3_ (0.2 mmol, 99.9 %, Alfa Aesar), 150.0 mg [BMIm]Cl (0.86 mmol, 98 %, Sigma Aldrich, dried under vacuum at 100 °C), and 450.0 mg AlCl_3_ (3.38 mmol, sublimed three times). The evacuated and sealed ampule was heated at 180 °C for 6 d and subsequently tilted and cooled to room temperature at Δ*T*/*t*=−6 K/h^−1^. The IL was decanted from the precipitated colorless AlCl_3_ and the deep red crystals of **1**, which were obtained in sizes of 0.03 to 1 mm. The crystals of **1** were identified visually, according to their color and shape, and separated mechanically from other crystalline species and most of the IL. No further treatment was applied to these crystals, as the small amounts of residual IL on the crystal surface did not impede the following investigations. The excess sodium cations were not detected in any precipitate and are assumed to remain dissolved in the IL. For syntheses with *M*=K, Rb, Cs, equivalent stoichiometric amounts of *M*Cl were used while all other parameters were the same as in the synthesis of **1**. In all three cases, excess metal cations crystallized as ternary *M*[AlCl_4_] compounds. For *M*=Rb, crystals of the target composition appeared only as a minor byproduct next to [BMIm](Bi_4_S_4_)[AlCl_4_]_5_.

**EDX Spectroscopy**: EDX measurements were conducted using a SU8020 (Hitachi) SEM equipped with a Silicon Drift Detector (SDD) X‐Max^N^ (Oxford) to check the chemical composition of the crystals. However, several problems impeded the interpretation of the measured data. The [AlCl_4_]^−^ ions partially decompose in the high‐energetic electron beam (*U*_a_=25 kV) that is necessary to activate bismuth for this measurement.^[45]^ Considering these factors, we were able to support the composition of Na(Bi_7_S_8_)[S(AlCl_3_)_3_]_2_[AlCl_4_]_2_ regarding the presence or absence of all elements. Calcd./exp. Na : Bi : S : Al : Cl (at.‐%) in **1**: 1.9 : 13.5 : 19.2 : 15.4 : 50/2(1) : 15(3) : 11(3) : 24(2) : 48(4). For other *M*, the following experimental ratios were determined: K : Bi : S : Al : Cl (at‐%): 1(1) : 14(2) : 19(3) : 18(2) : 48(6) ; Rb : Bi : S : A : Cl (at‐%): 2(1) : 15(2) : 17(3) : 21(2) : 47(5); Cs : Bi : S : Al : Cl (at‐%): 2(1) : 13(2) : 13(3) : 23(2) : 49(4).

**Powder X‐ray Diffraction**: Data collection was performed at 296(2) K with an X'Pert Pro MPD diffractometer (PANalytical) equipped with a Ge(220) hybrid‐monochromator using Cu‐K_α1_ radiation (*λ*=154.056 pm). Due to their sensitivity to moisture, the samples were contained in a glass capillary (Hilgenberg) with an outer diameter of 0.3 mm. A Le Bail fit was conducted for the lattice parameters of Na(Bi_7_S_8_)[S(AlCl_3_)_3_]_2_[AlCl_4_]_2_ based on the values gathered in the SCXRD measurements at 100(2) K with TOPAS.^[46]^

**X‐ray Crystal Structure Determination**: Single‐crystal X‐ray diffraction was measured on a four‐circle Kappa APEX II CCD diffractometer (Bruker) with a graphite(002)‐monochromator and a CCD‐detector at *T*=100(2) K. Mo‐*K*_α_ radiation (*λ*=71.073 pm) was used. The datasets were corrected for background, polarization and Lorentz factor using the APEX3 software suite.^[47]^ After integration,^[47]^ a numerical absorption correction based on an optimized crystal description was applied.^[48]^ The initial structure solution was performed with JANA2006^[49]^ and further refinement processed in SHELXL against *F*_o_^2^.[^50^, ^51^, ^52^] The unit cell setting was chosen so that (a) all angles are smaller than 90° and (b) one face of the cell is parallel to the layer packages in the structure, which also simplifies the group‐subgroup relationship to the silver compound.

Na(Bi_7_S_8_)[S(AlCl_3_)_3_]_2_[AlCl_4_]_2_: triclinic; *P* $\overset{-}{1}$ (no. 2); *T*=100(2) K; *a*=1846.4(1) pm, *b*=1905.1(2) pm, *c*=1065.5(2) pm, *α*=84.07(1)°, *β*=89.55(1)°, *γ*=49.68(1)°; *V*=2883.9(4)×10^6^ pm^3^; *Z*=2; *ρ*_*calcd*._=3.450 g cm^−3^; *μ*(Mo‐K_α_)=23.4 mm^−1^; 2*θ*_max_=54.0°, −23≤*h*≤23, −24≤*k*≤24, −13≤*l*≤13; 61896 measured, 12231 unique reflections, *R*_int_=0.087, *R*_σ_=0.098; 469 parameters, *R*_1_[7989 *F*_o_>4*σ*(*F*_o_)]=0.046, *wR*_2_(all *F*_o_^2^)=0.071, *GooF*=1.095, min./max. residual electron density: −3.87/5.67 e×10^−6^ pm^−3^. For atomic parameters see Table S2 of the Supporting Information.

Further details of the crystal structure determination are available from the Fachinformationszentrum Karlsruhe, D‐76344 Eggenstein‐Leopoldshafen (Germany), E‐mail: crysdata@fiz‐karlsruhe.de, on quoting the depository number CSD‐2090776 for Na(Bi_7_S_8_)[S(AlCl_3_)_3_]_2_[AlCl_4_]_2_.

## Conflict of interest

The authors declare no conflict of interest.

## Supporting information

As a service to our authors and readers, this journal provides supporting information supplied by the authors. Such materials are peer reviewed and may be re‐organized for online delivery, but are not copy‐edited or typeset. Technical support issues arising from supporting information (other than missing files) should be addressed to the authors.

Supporting Information
