# Supplementary material for: Ionothermal Synthesis of Sulfidobismuth spiro‐Dicubane Cations
Source: ChemistryOpen. 2021 Aug 13;11(2):e202100145. doi: 10.1002/open.202100145 (PMC8805384; doi:10.1002/open.202100145)
Supplement: Supplementary file 1 — Supporting Information [file OPEN-11-e202100145-s001.pdf]

ChemistryOpen

Supporting Information

Ionothermal Synthesis of Sulfidobismuth *spiro*-Dicubane Cations

Maximilian Knies and Michael Ruck*

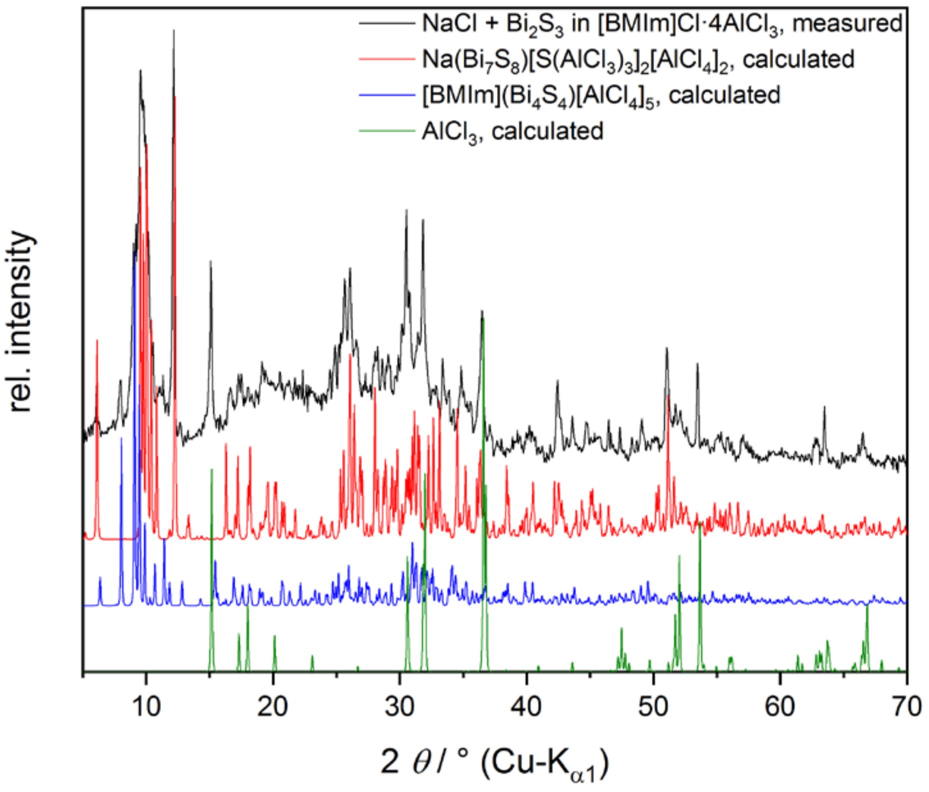

Figure S1. Powder X-ray diffractogram of the product of a synthesis targeting $Na(Bi_7S_8)[S(AlCl_3)_3]_2[AlCl_4]_2$. The high background, especially in the range 15° ≤ 2θ ≤ 35°, is caused by the glass capillary used for the air-sensitive sample.

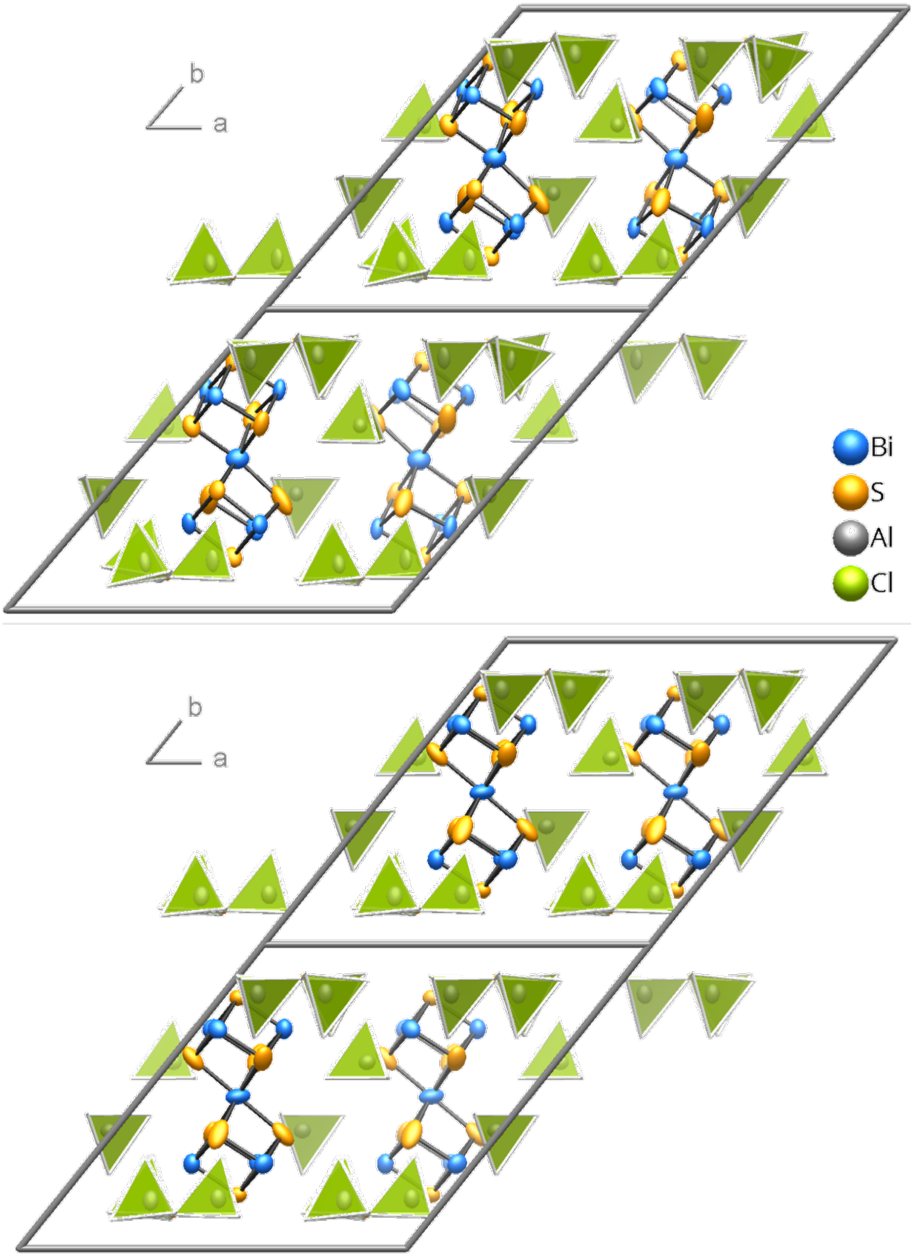

Figure S2. Crystal structure of $Na(Bi_7S_8)[S(AlCl_3)_3]_2[AlCl_4]_2$ at 100(2) K (top) and $Ag(Bi_7S_8)[S(AlCl_3)_3]_2[AlCl_4]_2$ in an "orthohexagonal" setting at 298(2) K (bottom). $[S(AlCl_3)_3]^{2-}$ and $[AlCl_4]^{-}$ anions are depicted as Al-centered polyhedra, Na^{+} and Ag^{+} cations are omitted for easier comparison. The ellipsoids comprise 99% (top) and 80% (bottom) of the probability density of the atoms.

Table S1. Interatomic distances *d* (/ pm) in $Na[Bi_7S_8][S(AlCl_3)_3]_2[AlCl_4]_2$.

atom pair		*d*	atom pair		*d*
Bi1	S1	266.3(3)	Bi7	S8	265.0(3)
Bi1	S3	259.3(3)	Bi7	Cl6	319.0(3)
Bi1	S4	261.1(3)	Bi7	Cl15	312.7(3)
Bi1	Cl2	314.7(3)	Bi7	Cl24	304.4(3)
Bi1	Cl10	339.9(3)	Na	Cl3	291.0(7)
Bi1	Cl11	316.9(3)	Na	Cl5	302.6(7)
Bi1	Cl21	308.7(3)	Na	Cl8	289.6(7)
Bi2	S1	264.5(3)	Na	Cl9	291.5(6)
Bi2	S2	254.3(4)	Na	Cl17	280.6(7)
Bi2	S4	263.7(3)	Na	Cl19	306.0(7)
Bi2	Cl7	344.4(3)	S9	Al1	227.4(5)
Bi2	Cl9	352.3(3)	S9	Al2	228.3(5)
Bi2	Cl12	310.4(3)	S9	Al3	227.2(5)
Bi2	Cl22	305.1(3)	S10	Al4	227.8(5)
Bi3	S1	265.3(3)	S10	Al5	226.5(5)
Bi3	S2	257.0(4)	S10	Al6	226.7(5)
Bi3	S3	265.1(3)	Al1	Cl1	210.0(5)
Bi3	Cl13	347.8(3)	Al1	Cl2	214.3(5)
Bi3	Cl14	312.7(3)	Al1	Cl3	213.8(5)
Bi3	Cl18	315.7(3)	Al2	Cl4	209.3(5)
Bi3	Cl25	307.1(3)	Al2	Cl5	215.2(5)
Bi4	S2	292.6(4)	Al2	Cl6	212.4(5)
Bi4	S3	272.4(4)	Al3	Cl7	211.0(5)
Bi4	S4	271.5(4)	Al3	Cl8	216.0(5)
Bi4	S5	288.2(4)	Al3	Cl9	214.0(5)
Bi4	S6	272.1(3)	Al4	Cl10	211.5(5)
Bi4	S7	290.4(4)	Al4	Cl11	212.4(5)
Bi5	S5	254.7(4)	Al4	Cl12	213.2(5)
Bi5	S6	263.3(3)	Al5	Cl13	211.2(5)
Bi5	S8	265.5(3)	Al5	Cl14	213.7(5)
Bi5	Cl1	343.1(3)	Al5	Cl15	213.9(5)
Bi5	Cl3	348.1(3)	Al6	Cl16	211.9(5)
Bi5	Cl8	317.0(3)	Al6	Cl17	215.1(5)
Bi5	Cl20	305.2(3)	Al6	Cl18	213.1(5)
Bi6	S6	262.1(3)	Al7	Cl19	213.7(5)
Bi6	S7	252.5(4)	Al7	Cl20	213.5(5)
Bi6	S8	266.7(3)	Al7	Cl21	213.0(5)
Bi6	Cl5	331.5(3)	Al7	Cl22	213.9(5)
Bi6	Cl16	347.7(3)	Al8	Cl23	212.0(5)
Bi6	Cl17	341.7(3)	Al8	Cl24	215.6(5)
Bi6	Cl26	306.7(3)	Al8	Cl25	214.3(5)
Bi7	S5	260.7(4)	Al8	Cl26	213.9(5)
Bi7	S7	259.4(4)			

Table S2. Coordinates, coefficients U_{ij} (/ 10 pm^2) of the tensors of the anisotropic displacement, and equivalent displacement parameters for the atoms in $Na[Bi_7S_8][S(AlCl_3)_3]_2[AlCl_4]_2$. U_{eq} is defined as one third of the trace of the orthogonalized U_{ij} tensor.

Atom	*x*	*y*	*z*	U_{11}	U_{22}	U_{33}	U_{23}	U_{13}	U_{12}	U_{eq}
Bi1	0.0682(1)	0.7102(1)	0.9021(1)	15(1)	17(1)	7(1)	–4(1)	3(1)	–11(1)	12(1)
Bi2	0.0325(1)	0.7267(1)	0.5479(1)	14(1)	30(1)	9(1)	–5(1)	2(1)	–16(1)	16(1)
Bi3	0.2198(1)	0.7310(1)	0.6803(1)	13(1)	24(1)	11(1)	–4(1)	3(1)	–14(1)	15(1)
Bi4	0.2636(1)	0.5020(1)	0.7296(1)	25(1)	22(1)	21(1)	–4(1)	3(1)	–14(1)	24(1)
Bi5	0.2816(1)	0.2863(1)	0.8108(1)	18(1)	24(1)	10(1)	1(1)	1(1)	–17(1)	15(1)
Bi6	0.4678(1)	0.2832(1)	0.9646(1)	16(1)	20(1)	13(1)	–5(1)	1(1)	–13(1)	15(1)
Bi7	0.4643(1)	0.2677(1)	0.6130(1)	26(1)	26(1)	13(1)	–9(1)	10(1)	–21(1)	18(1)
S1	0.0352(2)	0.8256(2)	0.7016(3)	14(2)	22(2)	10(2)	–5(2)	4(1)	–13(2)	14(1)
S2	0.2069(3)	0.6458(3)	0.5221(3)	20(2)	43(3)	22(2)	–18(2)	7(2)	–21(2)	26(1)
S3	0.2484(2)	0.6178(3)	0.8794(3)	16(2)	33(2)	12(2)	1(2)	–1(1)	–17(2)	20(1)
S4	0.0729(3)	0.6096(3)	0.7477(3)	25(2)	26(2)	11(2)	–5(2)	4(2)	–20(2)	18(1)
S5	0.2801(3)	0.3662(3)	0.6026(3)	27(2)	45(3)	18(2)	10(2)	–6(2)	–27(2)	28(1)
S6	0.2811(2)	0.3969(2)	0.9405(3)	17(2)	21(2)	11(2)	–4(2)	5(1)	–13(2)	15(1)
S7	0.4701(3)	0.3634(3)	0.7625(3)	52(3)	46(3)	19(2)	–12(2)	15(2)	–44(3)	29(1)
S8	0.4704(2)	0.1748(2)	0.8217(3)	22(2)	21(2)	11(2)	–7(2)	6(2)	–16(2)	16(1)
Na	0.2797(4)	0.0396(4)	0.7937(5)	50(4)	59(5)	27(4)	3(3)	–5(3)	–42(4)	41(2)
S9	0.3454(2)	0.1084(2)	0.3171(3)	12(2)	18(2)	7(2)	–5(1)	5(1)	–11(2)	11(1)
Al1	0.2453(3)	0.1713(3)	0.1390(3)	10(2)	21(3)	5(2)	–2(2)	2(2)	–13(2)	10(1)
Cl1	0.1688(2)	0.3132(2)	0.0819(3)	20(2)	18(2)	14(2)	2(1)	–1(1)	–11(2)	18(1)
Cl2	0.1510(2)	0.1445(2)	0.1826(3)	18(2)	29(2)	19(2)	1(2)	1(1)	–19(2)	19(1)
Cl3	0.3344(2)	0.0938(2)	–0.0063(3)	20(2)	22(2)	10(2)	–5(1)	4(1)	–13(2)	18(1)
Al2	0.4252(3)	0.1628(3)	0.3091(3)	17(2)	25(3)	4(2)	–1(2)	2(2)	–17(2)	13(1)
Cl4	0.3385(2)	0.3075(2)	0.2876(3)	24(2)	21(2)	30(2)	1(2)	–6(2)	–17(2)	24(1)
Cl5	0.5070(3)	0.1082(3)	0.1473(3)	33(2)	45(3)	13(2)	–11(2)	12(2)	–32(2)	24(1)
Cl6	0.5199(2)	0.0995(2)	0.4750(3)	18(2)	23(2)	14(2)	–2(1)	–3(1)	–14(2)	18(1)
Al3	0.2532(3)	0.1587(3)	0.4837(3)	16(2)	21(3)	6(2)	–5(2)	4(2)	–16(2)	12(1)
Cl7	0.1339(2)	0.3016(2)	0.4474(3)	19(2)	21(2)	20(2)	–4(2)	6(1)	–14(2)	19(1)
Cl8	0.3408(2)	0.1279(2)	0.6489(3)	22(2)	34(2)	8(2)	–6(2)	3(1)	–22(2)	18(1)
Cl9	0.2100(2)	0.0791(2)	0.5298(3)	19(2)	24(2)	15(2)	–5(1)	6(1)	–18(2)	16(1)
S10	0.1510(2)	0.8934(2)	0.1738(3)	15(2)	17(2)	10(2)	–4(1)	3(1)	–12(2)	13(1)
Al4	0.0773(3)	0.8329(3)	0.1848(3)	14(2)	23(3)	6(2)	–3(2)	4(2)	–15(2)	12(1)
Cl10	0.1709(2)	0.6875(2)	0.1839(3)	21(2)	22(2)	12(2)	–3(1)	1(1)	–16(2)	17(1)
Cl11	–0.0202(2)	0.8937(2)	0.0225(3)	20(2)	21(2)	14(2)	–3(1)	–2(1)	–14(2)	18(1)
Cl12	0.0001(2)	0.8777(3)	0.3497(3)	29(2)	35(2)	14(2)	–12(2)	14(2)	–26(2)	21(1)
Al5	0.2418(3)	0.8482(3)	0.3553(3)	15(2)	19(3)	8(2)	–5(2)	3(2)	–13(2)	12(1)
Cl13	0.3386(2)	0.7033(2)	0.4101(3)	18(2)	25(2)	15(2)	–2(2)	1(1)	–14(2)	20(1)
Cl14	0.1456(2)	0.9153(2)	0.4992(3)	26(2)	20(2)	12(2)	–8(1)	9(1)	–14(2)	20(1)
Cl15	0.3179(2)	0.8970(3)	0.3209(3)	29(2)	36(2)	18(2)	–5(2)	2(2)	–27(2)	22(1)
Al6	0.2403(3)	0.8477(3)	0.0048(3)	15(2)	20(3)	12(2)	–6(2)	5(2)	–14(2)	13(1)
Cl16	0.3542(2)	0.7025(2)	0.0187(3)	22(2)	23(2)	32(2)	–11(2)	15(2)	–16(2)	24(1)
Cl17	0.2990(2)	0.9153(2)	–0.0132(3)	20(2)	27(2)	16(2)	–4(2)	7(1)	–19(2)	18(1)
Cl18	0.1469(2)	0.8927(2)	–0.1579(3)	23(2)	36(2)	14(2)	–11(2)	5(2)	–21(2)	23(1)
Al7	0.0058(3)	0.6142(3)	0.2396(3)	15(2)	19(3)	10(2)	–3(2)	5(2)	–13(2)	13(1)
Cl19	–0.0912(2)	0.7624(2)	0.2059(3)	19(2)	14(2)	23(2)	–2(2)	4(2)	–9(2)	20(1)
Cl20	–0.0691(2)	0.5641(2)	0.2683(3)	21(2)	22(2)	35(2)	–3(2)	3(2)	–17(2)	24(1)

Cl21	0.0890(3)	0.5591(2)	0.0814(3)	30(2)	19(2)	18(2)	–7(2)	12(2)	–13(2)	24(1)
Cl22	0.0995(2)	0.5656(2)	0.4045(3)	20(2)	19(2)	21(2)	–1(2)	–4(2)	–12(2)	21(1)
Al8	0.4928(3)	0.3863(3)	0.2833(4)	12(2)	14(2)	15(2)	–3(2)	2(2)	–9(2)	13(1)
Cl23	0.5900(2)	0.2391(2)	0.3063(3)	16(2)	13(2)	18(2)	–2(1)	0(1)	–9(2)	16(1)
Cl24	0.3973(2)	0.4374(2)	0.4326(3)	21(2)	19(2)	21(2)	–5(2)	10(2)	–12(2)	21(1)
Cl25	0.5663(2)	0.4384(2)	0.2920(3)	15(2)	17(2)	30(2)	–3(2)	5(2)	–11(2)	20(1)
Cl26	0.4088(2)	0.4433(2)	0.1066(3)	26(2)	18(2)	18(2)	–2(2)	–3(2)	–11(2)	23(1)

Table S3. Coordinates of the *spiro*-atom in $Na(Bi_7S_8)[S(AlCl_3)_3]_2[AlCl_4]_2$ and in the "orthohexagonal" setting of $Ag(Bi_7S_8)[S(AlCl_3)_3]_2[AlCl_4]_2$.

**Atom**	***x***	***y***	***z***
Bi4(Na)	0.2636(1)	0.5020(1)	0.7296(1)
Bi4(Ag)	0.25	0.5	0.75
